# Associations between physical exercise and aging anxiety among older adults: a serial statistical mediation analysis involving self-efficacy and health anxiety

**DOI:** 10.3389/fpubh.2026.1929542

**Published:** 2026-08-26

**Authors:** Wenmin Yu, Guoxi Ye

**Affiliations:** 1 Department of Physical Education, Qingdao Technological University, Linyi, China; 2 Department of Physical Education, Shenzhen Polytechnic University, Shenzhen, China

**Keywords:** active aging, aging anxiety, health anxiety, older adults, physical exercise, self-efficacy, serial mediation model

## Abstract

**Background:**

With the acceleration of global population aging, mental health issues among older adults have become increasingly prominent. Aging anxiety, defined as apprehension regarding the aging process and associated physiological, psychological, and social role losses, significantly impacts the quality of life and health of the older adults. Physical exercise has been shown to be associated with better physical and mental health of older adults, yet the theoretical psychological mechanisms underlying their association remain insufficiently clarified. Therefore, this study aims to construct and test a serial mediation model to explore the statistical sequential indirect associations linking physical exercise to aging anxiety via self-efficacy and health anxiety.

**Methods:**

This study employed a cross-sectional survey design, targeting Chinese adults aged 60 and above. A total of 721 valid questionnaires were collected. Data were gathered using the *Physical Activity Rating Scale (PARS-3)*, the Aging Anxiety Scale, the General Self-Efficacy Scale, and the Health Anxiety Scale. Data analysis was performed using SPSS 27.0 and PROCESS 4.0 software. Common method bias tests, correlation analyses, multiple linear regression, and Bootstrap mediation effect tests were conducted to examine the relationships among physical exercise, self-efficacy, health anxiety, and aging anxiety, as well as the mediating pathways.

**Results:**

Correlation analysis revealed that physical exercise was significantly negatively correlated with aging anxiety (*r* = −0.232, *p* < 0.001), significantly positively correlated with self-efficacy (*r* = 0.225, *p* < 0.001), and significantly negatively correlated with health anxiety (*r* = −0.193, *p* < 0.001). Aging anxiety was negatively correlated with self-efficacy (*r* = −0.474, *p* < 0.001) and positively correlated with health anxiety (*r* = 0.483, *p* < 0.001); self-efficacy and health anxiety were significantly negatively correlated (*r* = −0.420, *p* < 0.001). Mediation analysis further revealed that the total effect of physical exercise on aging anxiety was −0.0525 (95% CI: [−0.0685, −0.0364]); the direct effect was −0.0221 (95% CI: [−0.036, −0.0081]), accounting for 42.10% of the total effect, and the total statistical indirect effect was −0.0304 (95% CI: [−0.040, −0.0216]), accounting for 57.90% of the total effect. The independent statistical indirect effect of self-efficacy was the most prominent (indirect effect = −0.0159, 95% CI: [−0.022, −0.0105]; accounting for 30.28% of the total effect). The indirect effect through health anxiety alone was −0.0078 (95% CI: [−0.0143, −0.0023]; 14.86%), and the serial indirect effect was −0.0067 (95% CI: [−0.0095, −0.0044]; 12.76%).

**Conclusion:**

This study supports that physical exercise is not only directly associated with lower aging anxiety in older adults but also shows significant statistical indirect associations through a potential serial pathway involving self-efficacy and health anxiety. Self-efficacy, as a crucial psychological resource, exhibits the strongest statistical indirect effect between physical exercise and aging anxiety. The findings deepen the understanding of the statistical association patterns through which physical exercise is linked to better mental health in older adults. They provide a theoretical basis and practical reference for designing targeted exercise interventions to mitigate aging anxiety among the older adults.

## 1 Introduction

With the acceleration of global population aging, the mental health of older adults has increasingly become a core issue of concern in public health and social policy domains. Among various psychological challenges, aging anxiety—defined as apprehension and fear regarding the aging process and associated losses in physiological, psychological, and social roles—significantly affects the quality of life and overall health of the older adults (1). Therefore, exploring effective intervention pathways to correlate with lower levels of aging anxiety in older adults holds substantial practical and theoretical significance.

Physical exercise, as a proactive and modifiable lifestyle factor, has been extensively supported by empirical research to be associated with multiple benefits in physical and mental health outcomes (2). For older adults, regular physical activity is associated with better physiological functions and slower cognitive decline and has also been shown to be linked to more positive mood states and higher psychological resilience (3). However, the potential correlational psychological pathways linking physical exercise and aging anxiety remain insufficiently clarified. Existing studies predominantly focus on examining direct effects or the role of a single mediating variable. There is a lack of systematic, integrated analysis concerning the complex psychological transmission pathways, particularly the sequential mechanisms involving multiple mediators.

From the perspectives of Social Cognitive Theory and health psychology, self-efficacy and health anxiety may be two key psychological variables linking physical exercise and aging anxiety. Self-efficacy refers to an individual’s belief in their capability to successfully execute specific behavioral tasks and is considered a core driver of behavioral change and maintenance (4). Theoretically, physical exercise may correlate with elevated self-efficacy, possibly via mastery experience, vicarious learning and positive feedback (this theoretical deduction requires causal experimental verification), vicarious learning, and positive feedback (5), which in turn may be linked to lower health anxiety. Health anxiety, characterized by an excessive worry and catastrophic interpretation of one’s health status, is closely related to aging anxiety as it often focuses on somatic symptoms and functional changes associated with aging (6). Thus, the theoretical model proposes that Based on existing theories, physical exercise may show sequential statistical correlations with elevated self-efficacy, reduced health anxiety, and concomitantly lower aging anxiety. This hypothesized serial mediation provides a coherent theoretical framework for understanding the internal processes through which physical exercise exerts its psychological benefits.

In light of this, the present study aims to construct a serial mediation model to systematically examine the serial statistical indirect correlational patterns of self-efficacy and health anxiety within the physical exercise–aging anxiety association among older adults. By elucidating this pathway, this study expects to theoretically deepen the understanding of the mechanisms through which physical exercise influences the mental health of the older adults and address the current research gap regarding the investigation of multiple mediating pathways.

## 2 Literature review and hypotheses

### 2.1 Physical exercise and aging anxiety in older adults

Aging anxiety refers to the worry, fear, or negative anticipation arising from an individual’s perception of their own aging and related physiological, psychological, and social role changes (7). It significantly impacts the quality of life and overall well-being of older adults (8). From the perspective of physiological mechanisms, regular physical exercise can effectively promote cardiovascular health, enhance musculoskeletal function, improve neuroendocrine regulation, and potentially delay the progression of various age-related chronic diseases. These physiological benefits not only directly improve the physical functioning and health perception of older adults but may also reduce the perceived vulnerability to negative aging outcomes such as disease and disability. Consequently, this can correlate with lower levels of anxiety experiences rooted in concerns about physical health decline.

Regarding psychosocial and socio-cognitive pathways, many physical exercise activities, particularly group- or community-based ones, constitute important opportunities for social participation. This participation provides a social support network, reduces feelings of social isolation (9), and may help older adults reconstruct a positive aging identity within peer groups. It can also shift excessive focus away from aging-related concerns, thereby buffering aging anxiety at psychological and social levels. Based on this, the present study proposes the following hypothesis:

*H1*: Physical exercise shows a significant negative correlational link with aging anxiety among older adults.

### 2.2 The mediating role of self-efficacy

Self-efficacy, referring to an individual’s belief in their capability to execute specific tasks or cope with particular situations, is formed and altered primarily through direct mastery experiences, vicarious experiences, verbal persuasion, and physiological/affective states. Physical exercise, as a repeatable and modifiable behavioral practice, provides participants with ongoing direct mastery experiences. When individuals achieve set goals or overcome physical or mental challenges through exercise, these successful experiences serve as a powerful means to reinforce their belief in their capabilities (10). Simultaneously, observing the progress of others during exercise and receiving encouragement from coaches or peers can be important channels for enhancing self-efficacy (11).

According to Bandura’s Social Cognitive Theory, high self-efficacy can increase an individual’s motivation and resilience when facing challenges. It promotes the adoption of active coping strategies, thereby buffering negative emotional responses in stressful situations (12). Aging anxiety, a common psychological state in old age, primarily manifests as worry and fear regarding a series of potential threats associated with the aging process. These threats include physical decline, loss of social roles, decreased cognitive ability, and the proximity of death. Theoretically, when older adults have strong confidence in their ability to cope with aging-related challenges, their perceived uncontrollability and threat appraisal of the aging process may decrease accordingly. High self-efficacy correlates with lower aging anxiety, and theories propose several potential correlational pathways to explain this co-occurrence. These include enhancing problem-focused coping abilities, facilitating adaptive emotion regulation, and strengthening the psychological sense of control over the aging process (13). Based on this, the study proposes the following hypotheses:

*H2*: Physical exercise is positively correlated with self-efficacy in older adults.

*H3*: Self-efficacy displays a significant negative correlation with aging anxiety.

*H4*: Self-efficacy carries a significant independent indirect correlational link between physical exercise and aging anxiety.

### 2.3 The mediating role of health anxiety

From a psychophysiological mechanism perspective, long-term, moderate physical exercise can effectively enhance cardiorespiratory function, muscle strength, and neuroendocrine regulation in older adults. These physiological benefits directly improve their baseline health status and somatic perception. Consequently, this reduces sensitivity to anxiety triggered by physical functional decline or ambiguous symptoms (14). Analyzing the socio-cognitive and psychological pathways, physical exercise, as a proactive health behavior, can strengthen older adults’ sense of self-efficacy and control. This fosters greater confidence in managing their own health. Furthermore, group-based or outdoor activities provide social support and positive emotional experiences. These help divert excessive attention away from illness and break the vicious cognitive cycle of “bodily sensation – catastrophic interpretation.”

Health anxiety typically involves the catastrophic misinterpretation of bodily sensations and persistent fear of disease. This pattern of hypervigilance and negative cognitive appraisal may transfer to the individual’s evaluation of “aging”—a natural physiological process. Normal age-related changes may be misconstrued as signs of functional loss or decline, thereby increasing overall anxiety about aging (15). Secondly, according to stress and coping theory, health issues are a core stressor for older adults. Persistent anxiety about health constitutes a form of chronic psychological stress. This can deplete an individual’s psychological resources and weaken their adaptive capacity to cope with aging-related challenges, potentially leading to more generalized aging anxiety. Additionally, the life-span developmental perspective suggests that health often holds core value in old age, serving as the foundation for maintaining autonomy and meaning in life. Therefore, anxiety about health threats can easily generalize into deeper concerns about the loss of personal value, future uncontrollability, and the end of life—core dimensions of aging anxiety.

Thus, physical exercise may help improve the physical health, bodily function, and self-efficacy of older adults. This could, in turn, reduce their excessive worry and negative expectations regarding their health status, thereby alleviating health anxiety. Health anxiety, as an individual’s cognitive and emotional response to perceived health threats, has been confirmed to be closely related to aging anxiety. When health anxiety is mitigated, older adults’ worries about potential issues accompanying the aging process—such as functional decline, loss of social roles, and limitations on life meaning—may also diminish. Based on this, the study proposes the following hypotheses:

*H5*: Higher levels of physical exercise are associated with lower levels of health anxiety in older adults.

*H6*: Health anxiety shows a significant positive correlation with aging anxiety.

*H7*: Health anxiety carries a significant independent indirect correlational link between physical exercise and aging anxiety.

### 2.4 The serial mediating role of self-efficacy and health anxiety

Self-efficacy is a core construct in Social Cognitive Theory. In the health domain, it often manifests as the strength of an individual’s belief in their ability to manage health, perform health behaviors, and cope with health threats. Individuals with high self-efficacy tend to adopt proactive health behaviors. They engage in reasonable filtering and interpretation of health information. When facing bodily discomfort or signs of illness, they demonstrate greater psychological resilience and adaptive coping strategies (16).

**Figure 1:**
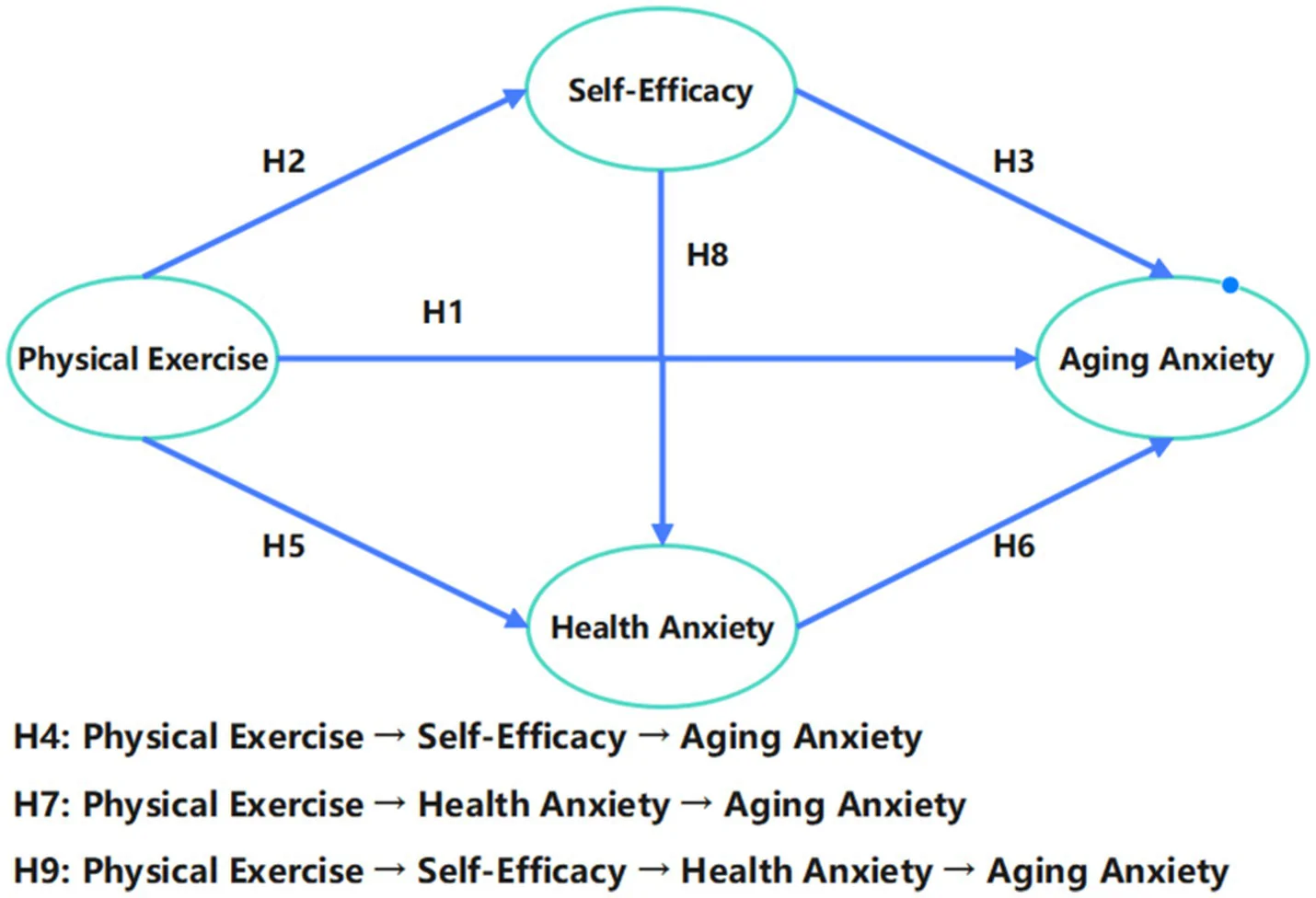
Research hypothesis model.

At the cognitive appraisal level, high self-efficacy can buffer individuals from catastrophizing potential health threats. It reduces excessive vigilance and misattribution regarding bodily sensations, thereby decreasing the activation of anxious emotions. At the behavioral regulation level, individuals with higher self-efficacy are more likely to persist with preventive health behaviors. This sense of controllability and agency helps mitigate anxiety arising from uncertainty (17). At the emotional regulation level, self-efficacy is closely linked to positive coping resources, which can effectively correlate with lower levels of anxiety responses in stressful situations. In summary, self-efficacy can be regarded as a key psychological resource. It systematically and inhibitively influences health anxiety by shaping an individual’s patterns of cognitive, behavioral, and emotional responses (Figure 1).

Physical exercise has been confirmed to have a positive effect on alleviating aging anxiety, yet its underlying mechanisms require further exploration. Integrating perspectives from Social Cognitive Theory and health psychology, self-efficacy—an individual’s belief in their capability to perform specific behaviors—may play a crucial mediating role between physical exercise and mental health. Simultaneously, health anxiety—excessive worry about one’s health status—may mediate the relationship for older adults facing the aging process. Specifically, physical exercise may enhance self-efficacy in older adults. This, in turn, may reduce their anxiety levels concerning health issues, ultimately lessening aging anxiety. Based on this, the study proposes the following research hypotheses:

*H8*: Self-efficacy is negatively correlated with health anxiety.

*H9*: Self-efficacy and health anxiety jointly form a significant serial indirect correlational pathway linking physical exercise to aging anxiety.

## 3 Research methods

### 3.1 Participants

The demographic analysis of the older adults population in this study strictly adhered to the World Health Organization’s age definition for older populations in developing countries, i.e., individuals aged 60 and above. To ensure the scientificity and representativeness of the sample, a multi-stage stratified random sampling method was adopted for participant recruitment, and the specific recruitment process, inclusion/exclusion criteria, and sampling strategy are detailed as follows:

Regarding the recruitment process, participants were recruited from three types of settings to cover diverse living environments of the older adults in China: urban and rural communities (42.2%, *n* = 304), older adults care centers (33.7%, *n* = 243), and community health clinics (24.1%, *n* = 174). The recruitment was carried out by trained researchers and community workers from March to June 2025. First, we contacted the management departments of the selected settings (community neighborhood committees, older adults care centers, and community health clinics) to obtain support and access to potential participants. Second, researchers introduced the purpose, content, and voluntary participation principle of the study to potential participants face-to-face, and distributed informed consent forms. Only those who voluntarily signed the informed consent form were included in the questionnaire survey. Finally, researchers guided participants to fill out the questionnaire on the spot; for older adults participants with poor eyesight or mobility, researchers read the questionnaire content aloud and recorded the answers truthfully to ensure the completeness and accuracy of the responses.

The inclusion criteria for participants were: (1) Aged 60 years and above, in line with the WHO’s age definition for the older adults in developing countries; (2) Clear consciousness, normal cognitive function, and able to understand and respond to the questionnaire content independently or with appropriate guidance; (3) Voluntarily participate in the study and sign the informed consent form; (4) Have a fixed residence in the surveyed area and have lived there for more than 6 months (to ensure familiarity with the local living environment and consistency of health status). The exclusion criteria were: (1) Severe cognitive impairment, mental illness, or language communication barriers that prevent effective response to the questionnaire; (2) Acute diseases or severe chronic diseases in the acute stage (e.g., severe heart failure, acute stroke) that display correlational links with the ability to participate in the survey; (3) Refusal to sign the informed consent form or withdrawal from the study halfway; (4) Incomplete basic information, unable to confirm age, gender, and other key demographic indicators.

In terms of sampling strategy, multi-stage stratified random sampling was used to ensure the representativeness of the sample. First, China was stratified into three major regions: Eastern, Central, and Western, based on economic development level and geographical location, which are the core stratification variables. Second, within each region, we stratified by urban and rural areas (second-level stratification) and by type of living environment (community, older adults care center, community health clinic, third-level stratification). Third, according to the proportion of the older adults population in each stratum (referring to the latest national census data), a random sampling method was used to determine the number of samples to be selected in each stratum, ensuring that the sample proportion of each stratum was consistent with the proportion of the older adults population in the corresponding region and environment. This sampling strategy effectively avoided sampling bias caused by single setting or regional concentration.

The age distribution of the sample was as follows: 60–64 years (25.9%, *n* = 187), 65–69 years (23.6%, *n* = 170), 70–74 years (24.8%, *n* = 179), and 75–80 years (25.7%, *n* = 185), indicating a relatively uniform distribution across age groups. A total of 750 questionnaires were distributed, with 732 returned and deemed valid initially, yielding a preliminary valid response rate of 97.6%. Following data collection, the responses were cleaned and screened based on the following criteria: First, incomplete questionnaires with missing key variables were excluded. Second, questionnaires exhibiting patterned responses (e.g., consecutive identical answers, wave-like patterns) were removed to ensure authenticity and conscientiousness. Finally, questionnaires from respondents not meeting the age criterion (≥60 years) were excluded. After this process, 11 invalid questionnaires were discarded, resulting in a final valid sample of 721 participants.

Regarding the representativeness of the sample: The sample covers three major regions (Eastern, Central, and Western China) with comparable proportions (Eastern: 33.7%, *n* = 243; Central: 33.4%, *n* = 241; Western: 32.9%, *n* = 237), which is consistent with the regional distribution of China’s older adults population. In terms of residence type, the sample includes 359 rural participants (49.8%) and 362 urban participants (50.2%), basically matching the urban–rural ratio of China’s older adults population. In addition, the sample is balanced in gender (males: 47.4%, *n* = 342; females: 52.6%, *n* = 379), age groups, and chronic disease status (with chronic diseases: 49.7%, *n* = 358; without chronic diseases: 50.3%, *n* = 363), covering older adults people from different living environments (communities, older adults care centers, clinics). Therefore, the sample has a certain degree of representativeness and can reflect the characteristics of the broader older adults population in China to a certain extent. However, it should be noted that due to the limitations of sampling scope and practical conditions, the sample cannot fully represent all older adults groups in China (e.g., older adults people in remote pastoral areas are not included), and this limitation is discussed in the discussion section of the paper.

Overall, the sample demonstrated a well-balanced distribution across key demographic variables. This balance helps control for potential confounding effects of these covariates in the subsequent analysis of the “physical exercise → self-efficacy → health anxiety → aging anxiety” serial mediation mechanism, thereby enhancing the reliability and generalizability of the study findings (Table 1).

**Table 1:** Demographic characteristics of the survey participants (*N* = 721).

Variable	Category	Frequency	Percentage (%)
Age	60–64	187	25.9
	65–69	170	23.6
	70–74	179	24.8
	75–80	185	25.7
Gender	Male	342	47.4
	Female	379	52.6
Region	Eastern	243	33.7
	Central	241	33.4
	Western	237	32.9
Residence	Rural	359	49.8
	Urban	362	50.2
Chronic disease status	No	363	50.3
	YES	358	49.7

### 3.2 Measurement instruments

#### 3.2.1 Physical activity rating scale (PARS-3)

The revised Physical Activity Rating Scale (PARS-3)* by Liang (18) was used to assess participants’ physical activity level. This scale evaluates exercise volume based on three dimensions: exercise intensity, frequency, and duration, rated on a 5-point Likert scale. The total exercise volume score was calculated using the formula: Exercise Volume = Frequency × (Duration − 1) × Intensity. Scores range from 0 to 100, with higher scores indicating greater activity levels. Based on the total score, participants were categorized into three levels: low activity (≤19 points), moderate activity (20–42 points), and high activity (43–100 points). In this study, the scale showed good structural validity with a Kaiser–Meyer–Olkin (KMO) measure of 0.700 and Bartlett’s test of sphericity value of 560.689 (*p* < 0.001). Its reliability was satisfactory, with a Cronbach’s *α* coefficient of 0.769.

#### 3.2.2 Aging anxiety scale

Aging anxiety was measured using the Aging Anxiety Scale developed by Ravary et al. (19). The scale consists of 7 items rated on a 5-point Likert scale. Total scores range from 7 to 35, with higher scores indicating greater aging anxiety. This scale was translated and validated by Gou et al. (20), which verified its applicability for assessing aging anxiety among older adults within the Chinese sociocultural context. In this study, the scale demonstrated good structural validity (KMO = 0.844; Bartlett’s test = 762.352, *p* < 0.001) and acceptable reliability (Cronbach’s *α* = 0.738).

#### 3.2.3 General self-efficacy scale

Self-efficacy was assessed using the General Self-Efficacy Scale (21). The scale comprises 10 items rated on a 5-point Likert scale, with total scores ranging from 10 to 50. Higher scores indicate stronger self-efficacy. In this study, the scale exhibited excellent structural validity (KMO = 0.904; Bartlett’s test = 1430.168, *p* < 0.001) and good internal consistency (Cronbach’s *α* = 0.812).

#### 3.2.4 Health anxiety scale

Health anxiety was measured using the short-form Health Anxiety Scale originally developed by Salkovskis (22) and later translated into Chinese by Zhang et al. (23). The scale consists of 18 items rated on a 5-point Likert scale and assesses two dimensions: perceived likelihood of illness and fear of negative consequences of illness. In this study, the scale showed excellent structural validity (KMO = 0.929; Bartlett’s test = 2091.889, *p* < 0.001) and good reliability (Cronbach’s *α* = 0.837).

### 3.3 Data processing and analysis

The collected data were analyzed using SPSS 27.0 and PROCESS 4.0 software. The statistical procedures included the following steps: (1) Classification, transformation, and computation of relevant valid data; (2) Assessment of common method bias using Harman’s single-factor test; (3) Examination of the reliability and construct validity of the measurement instruments by calculating Cronbach’s *α* coefficients and Bartlett’s test of sphericity; (4) Application of correlation analysis and linear regression to verify relationships among variables and to test the significant effects of physical exercise, self-efficacy, and health anxiety on aging anxiety in older adults; (5) Use of the Bootstrap method to examine the independent and serial mediating roles of self-efficacy and health anxiety in the relationship between physical exercise and aging anxiety in older adults.

## 4 Research results

### 4.1 Common method bias test

Common method bias was assessed using statistical methods in this study. Exploratory factor analysis was conducted on all measurement items via Harman’s single-factor test. The results indicated that the variance explained by the first unrotated principal component was 20.76%, which is below the critical threshold of 40%. This suggests that no severe common method bias was present in the data. Although a single factor did not account for the majority of the variance, further measures were implemented during the questionnaire design phase to enhance the robustness of the findings and control for potential bias. These measures included: (1) administering the questionnaires anonymously; (2) separating the item order for independent and dependent variables; and (3) employing scales from multiple sources while ensuring item clarity. Subsequent analyses could also employ supplementary methods, such as controlling for unmeasured latent method factors or incorporating marker variables for additional verification. In summary, the quality of the data in this study meets the fundamental requirements for statistical analysis.

### 4.2 Correlation analysis

To examine the relationships among physical exercise, aging anxiety, self-efficacy, and health anxiety, this study presents the descriptive statistics and correlation analysis for these variables in Table 2. Table 2 displays the means (*M*), standard deviations (SD), and Pearson correlation coefficients between the variables.

**Table 2:** Means, standard deviations, and correlation coefficients of the study variables.

Variable	*M*	SD	Physical exercise	Aging anxiety	Self-efficacy	Health anxiety
Physical exercise	27.85	21.834				
Aging anxiety	20.38	4.933	−0.232^**^			
Self-efficacy	35.58	5.963	0.225^**^	−0.474^**^		
Health anxiety	49.60	10.081	−0.193^**^	0.483^**^	−0.420^**^	

*p* < 0.05; ^*^p < 0.01; ^**^p < 0.001 (consistent across analyses).

In terms of mean scores, the average score for physical exercise was relatively low (*M* = 27.85, SD = 21.83), indicating a generally low frequency of physical activity among the older adults in the sample. The mean score for aging anxiety was 20.38 (SD = 4.93), at a moderate level, reflecting a certain degree of concern about the aging process among the participants. Self-efficacy showed a higher mean score (*M* = 35.58, SD = 5.96), suggesting that the participants had relatively strong confidence in coping with aging-related challenges. The mean score for health anxiety was 49.60 (SD = 10.08), also at a moderate level.

Correlation analysis results indicated that physical exercise was significantly negatively correlated with aging anxiety (*r* = −0.23, *p* < 0.001), significantly positively correlated with self-efficacy (*r* = 0.23, *p* < 0.001), and significantly negatively correlated with health anxiety (*r* = −0.19, *p* < 0.001). Aging anxiety was significantly negatively correlated with self-efficacy (*r* = −0.47, *p* < 0.001) and significantly positively correlated with health anxiety (*r* = 0.48, *p* < 0.001). Self-efficacy and health anxiety also showed a significant negative correlation (*r* = −0.42, *p* < 0.001). All correlation coefficients reached statistical significance (*p* < 0.001), indicating associations of moderate strength among the variables and providing preliminary support for further investigation of mediation effects.

This study employed multiple linear regression analysis to examine the combined predictive effects of physical exercise, self-efficacy, and health anxiety on aging anxiety in older adults. The results indicated that the overall regression model was statistically significant (*p* < 0.001), suggesting that these variables collectively explain variance in aging anxiety. Specifically, physical exercise demonstrated a significant negative predictive effect on aging anxiety (*B* = −0.022, *β* = −0.098, *p* < 0.01). This indicates that higher levels of physical exercise are associated with lower levels of aging anxiety. Simultaneously, self-efficacy also showed a significant negative predictive effect (*B* = −0.258, *β* = −0.312, *p* < 0.001), meaning individuals with stronger self-efficacy reported lower levels of aging anxiety. Conversely, health anxiety exhibited a significant positive predictive effect on aging anxiety (*B* = 0.163, *β* = 0.333, *p* < 0.001). That is, higher levels of health anxiety were associated with more pronounced aging anxiety.

Furthermore, the tolerance for each predictor variable was greater than 0.8, and the variance inflation factor (VIF) for each was below 1.3. This indicates the absence of severe multicollinearity in the model, enhancing the robustness of the analytical results.

In summary, physical exercise, self-efficacy, and health anxiety are all significant influencing factors of aging anxiety, with self-efficacy and health anxiety showing stronger predictive effects. These findings provide a basis for further exploration of the mediating mechanisms among these variables (Table 3).

**Table 3:** Multiple regression analysis of physical exercise, self-efficacy, and health anxiety on aging anxiety in older adults.

Variable	Aging anxiety in older adults					
	*B*	SE	*β*	*T*	Tolerance	VIF
Constant	22.101	1.538		14.366		
Physical exercise	−0.022	0.007	−0.098	−3.095	0.937	1.067
Self-efficacy	−0.258	0.028	−0.312	−9.149	0.802	1.247
Health anxiety	0.163	0.017	0.333	9.831	0.814	1.229

### 4.3 Mediation effect tests

Table 4 presents the results of the mediation pathway analysis examining the effect of physical exercise on aging anxiety in older adults. The results show that the total effect of physical exercise on aging anxiety was −0.0525 (95% CI: −0.0685, −0.0364), indicating that physical exercise significantly and negatively predicts aging anxiety. Further analysis revealed that this total effect can be decomposed into a direct effect and a mediation effect. The direct effect was −0.0221 (95% CI: −0.036, −0.0081), accounting for 42.10% of the total effect. The total mediation effect was −0.0304 (95% CI: −0.040, −0.0216), accounting for 57.90% of the total effect. This indicates that self-efficacy and health anxiety play significant mediating roles in the relationship between physical exercise and aging anxiety.

**Table 4:** Mediation pathway analysis via bootstrap testing.

Effect	Path	Effect size	Standard error	LLCI	ULCI	Percentage of total effect
Total effect	Direct path	−0.0525	0.0082	−0.0685	−0.0364	100.00
Direct effect		−0.0221	0.0071	−0.036	−0.0081	42.10
Total indirect effect		−0.0304	0.0047	−0.040	−0.0216	57.90
Indirect effect	Pathway 1	−0.0159	0.0029	−0.022	−0.0105	30.28
	Pathway 2	−0.0078	0.0031	−0.0143	−0.0023	14.86
	Pathway 3	−0.0067	0.0013	−0.0095	−0.0044	12.76

Within the mediation effects, three specific significant pathways were identified:

Pathway 1 (Physical Exercise → Self-Efficacy → Aging Anxiety): The effect size was −0.0159 (95% CI: −0.022, −0.0105), accounting for 30.28% of the total effect. This statistical indirect pathway indicates that higher physical exercise is associated with higher self-efficacy, which in turn is associated with lower aging anxiety, underscoring self-efficacy as the most important theoretical mechanism that requires further longitudinal validation.

Pathway 2 (Physical Exercise → Health Anxiety → Aging Anxiety): The effect size was −0.0078 (95% CI: −0.0143, −0.0023), accounting for 14.86% of the total effect. This path suggests that physical exercise can also indirectly reduce aging anxiety by lowering health anxiety levels, highlighting the mediating role of health anxiety in this process.

Pathway 3 (Physical Exercise → Self-Efficacy → Health Anxiety → Aging Anxiety): The effect size was −0.0067 (95% CI: −0.0095, −0.0044), accounting for 12.76% of the total effect. This is a serial mediation path, indicating that self-efficacy not only directly affects aging anxiety but can also further transmit the positive effects of physical exercise by alleviating health anxiety. It demonstrates the sequential mediating roles of self-efficacy and health anxiety.

**Figure 2:**
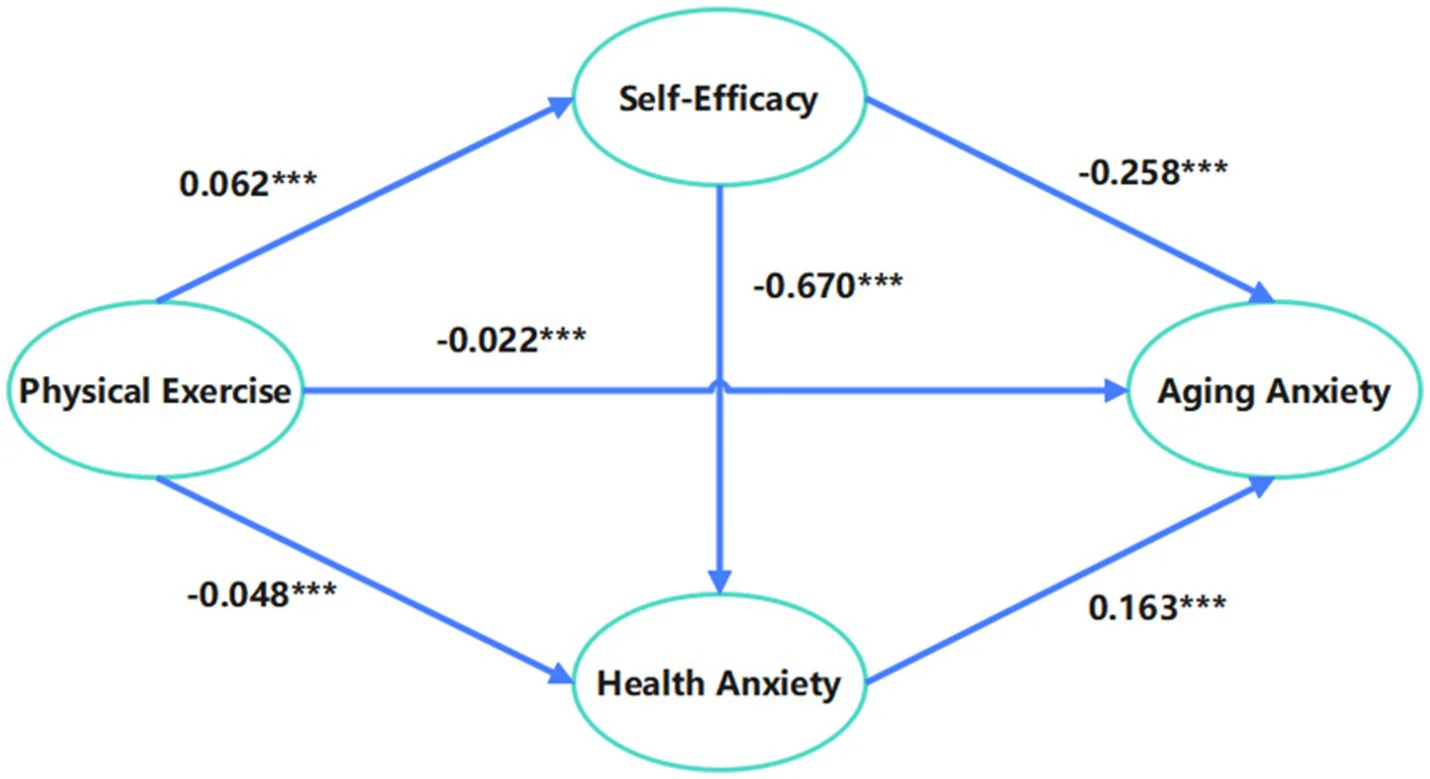
Serial mediation model.

In summary, this study supports that physical exercise is not only directly associated with lower aging anxiety in older adults but also exerts significant statistical indirect effects through both the independent and serial pathways involving self-efficacy and health anxiety. Among these, the statistical indirect effect of self-efficacy is particularly prominent, serving as a key theoretical psychological mechanism for understanding the association between physical exercise and aging anxiety (Figure 2).

## 5 Discussion

### 5.1 The correlate with of physical exercise on aging anxiety in older adults

All findings in this section reflect cross-sectional correlations and cannot confirm causal effects. Consistent with prior research, physical exercise shows a significant negative correlation with aging anxiety, which lays a theoretical foundation for discussing potential correlational pathways linking lifestyle and late-life mental health. Aging anxiety, defined as negative cognitions and emotions surrounding age-related physiological, psychological, and social losses, arises from multiple interacting factors. As a modifiable lifestyle factor, physical exercise correlates with lower aging anxiety, and existing theories propose integrated physiological, psychological, and social correlational pathways to interpret this co-occurrence.

Physiologically, regular exercise improves cardiovascular and musculoskeletal function, enhances balance and neuroplasticity, and modulates endocrine activity to increase endorphin and serotonin release—changes that directly correlate with lower levels of physical decline and correlate with lower levels of stress-related anxiety (24). Cognitively, preserved physical function challenges the negative schema equating aging with inevitable disability, reshaping older adults’ subjective experience of aging at its core.

Theoretically, physical exercise aligns with multidimensional successful aging criteria by promoting physical health, cognitive maintenance, and social engagement. Framed as an active coping strategy for the developmental stressor of aging, it simultaneously reduces stressor intensity via physiological improvements and enhances coping capacity through psychological and social resource accumulation, creating a self-reinforcing positive cycle.

### 5.2 The mediating role of self-efficacy

This study confirms the significant independent indirect effect of self-efficacy between physical exercise and aging anxiety, advancing our theoretical understanding of how physical activity fosters generalized psychological resources that buffer age-related negative cognitions and emotions.

This statistical indirect correlational pathway reveals a theoretical co-occurrence pattern linking physical activity and psychological perceptions; the causal transformation process requires longitudinal verification. Grounded in Bandura’s Social Cognitive Theory, self-efficacy—defined as beliefs in one’s ability to execute goal-directed behaviors—develops through mastery experiences, vicarious learning, verbal persuasion, and physiological/affective states (25). Regular exercise systematically activates all four sources: goal achievement provides direct mastery experiences; peer progress offers vicarious models; social encouragement delivers verbal persuasion; and improved energy and sleep generate positive physiological feedback. This process transforms exercise into a platform for building self-efficacy that generalizes beyond physical activity to broader aging-related challenges, reducing feelings of powerlessness and anxiety by fostering a sense of “I can adapt to change.”

Theoretically, this finding highlights the central agentic role of self-efficacy in successful aging, which encompasses disease avoidance, functional maintenance, life engagement, and psychological adaptation (26). High self-efficacy leads older adults to appraise aging challenges as manageable rather than uncontrollable, promoting proactive coping strategies such as health information seeking and social role adaptation. By enhancing self-efficacy, exercise strengthens older adults’ sense of agency over their aging process, aligning with modern gerontological principles of active aging and empowerment.

Notably, self-efficacy holds unique significance in later life. Retirement, reduced social roles, and physical decline often erode efficacy sources rooted in occupational or familial status (27). Exercise, as a controllable activity with immediate feedback, provides a novel, achievement-based domain for rebuilding efficacy independent of past social standing. This bodily-derived confidence serves as a distinct buffer against existential anxiety stemming from lost social comparisons and functional decline.

### 5.3 The mediating role of health anxiety

This study verifies the significant independent mediating effect of health anxiety, establishing a critical link between physical practice, health-specific cognitions, and global aging-related emotional experiences.

This mediating pathway reflects a cognitive mechanism whereby exercise reduces aging anxiety by reshaping health-related appraisals. Aging anxiety is fundamentally rooted in perceptions of age-related losses, with health and functional decline representing the most salient and direct threat (1). Cognitive-behavioral theory posits that anxiety arises from excessive attention to, and catastrophic interpretation of, potential threats (28). For older adults, normal bodily changes or chronic disease symptoms are often misinterpreted as signs of impending functional collapse, triggering health anxiety that can generalize into pervasive fear of the entire aging process (29).

Exercise intervenes at the cognitive appraisal stage by providing objective evidence of preserved bodily function (30) and positive somatic feedback (e.g., post-exercise relaxation, improved mood). These sustained, controllable bodily experiences shift cognitive patterns from catastrophizing minor discomforts to rational interpretation of bodily signals, reducing hypervigilance and catastrophic thinking. When the core belief of “my body is irreversibly declining” is challenged, global aging anxiety naturally diminishes.

Health anxiety exerts a particularly strong influence in later life, when chronic conditions, frequent bodily discomfort, and mortality salience elevate health concerns to a central life domain. Interventions targeting health anxiety thus produce multiplier effects on overall aging anxiety. As a self-directed, non-pharmacological health management strategy, exercise fosters a sense of active control over health outcomes, counteracting the passivity and helplessness that often accompany illness and aging.

### 5.4 The serial mediating role of self-efficacy and health anxiety

This study confirms the significant serial indirect pathway of physical exercise → self-efficacy → health anxiety → aging anxiety, which aligns with the theoretical sequence of physical practice → psychological resource accumulation → cognitive reappraisal → emotional regulation.

The serial model integrates Social Cognitive Theory, cognitive anxiety models, and successful aging theory: self-efficacy serves as the critical bridge between health behavior and anxious cognitions, while reduced health anxiety reflects cognitive reappraisal applied to aging-related experiences. This pathway reveals that successful aging extends beyond disease avoidance and functional maintenance to include the construction of an empowered psychological self-schema through sustained behavioral engagement—a schema that enables rational filtering and effective coping with inevitable age-related challenges (31).

Compared to single-mediator models, the serial pathway clarifies the layered, indirect nature of exercise’s mental health benefits. Psychological improvements do not arise directly from physical activity alone but require sequential cultivation of internal resources and modification of core cognitions. This finding underscores that exercise interventions targeting aging anxiety will have limited or unsustainable effects if they fail to address these underlying psychological mechanisms.

## 6 Conclusions and future directions

### 6.1 Conclusion

This study, centered on the core question of “how physical exercise influences aging anxiety in older adults,” constructed and tested a theoretical model with self-efficacy and health anxiety as serial mediators, based on perspectives from Social Cognitive Theory and health psychology. Through a questionnaire survey and data analysis involving 721 older adults, the following main conclusions were drawn:

1. Physical exercise displays a significant negative statistical correlation with aging anxiety among older adults: elders reporting higher physical activity levels tend to report less severe aging anxiety, yet this co-occurrence does not confirm that physical activity causally reduces aging anxiety.
2. Self-efficacy carries a significant independent statistical indirect correlational link between physical exercise and aging anxiety. The cross-sectional co-occurrence pattern shows that greater physical activity correlates with stronger self-efficacy, and stronger self-efficacy further correlates with milder aging anxiety. This statistical indirect association provides a theoretical correlational interpretation for the link between exercise and aging anxiety, rather than proving a causal transmission process.
3. Health anxiety plays an independent mediating role between physical exercise and aging anxiety. Physical exercise can effectively correlate with lower levels of health anxiety, and health anxiety positively predicts aging anxiety, with a significant mediating effect. This indicates that physical exercise indirectly reduces overall aging anxiety by decreasing excessive worry about personal health among older adults.
4. Self-efficacy and health anxiety form a serial mediation pathway. Self-efficacy significantly negatively influences health anxiety, and physical exercise further alleviates aging anxiety through the sequential pathway of “enhancing self-efficacy → reducing health anxiety.” This serial mediation model reveals the progressive psychological mechanism through which physical exercise influences aging anxiety: physical exercise first enhances an individual’s self-efficacy, which then reduces anxiety about health issues, ultimately lowering overall anxiety regarding the aging process.

In summary, this study not only supports the significant negative association between physical exercise and aging anxiety in older adults but also systematically provides preliminary evidence for the underlying theoretical mechanisms: physical exercise is directly associated with lower aging anxiety, and it also shows significant statistical indirect effects through its associations with higher self-efficacy and lower health anxiety. Furthermore, self-efficacy and health anxiety form a significant serial statistical indirect pathway.

### 6.2 Research limitation

While this study, by constructing a serial mediation model, reveals the psychological mechanisms through which physical exercise influences aging anxiety in older adults and contributes to a deeper theoretical understanding, it still possesses several limitations that need to be addressed in future research.

First, the cross-sectional design precludes any causal inference. This study employed a questionnaire survey to collect data at a single time point. Although the theoretical construction and statistical analyses revealed statistical associations and indirect effects among the variables, they cannot confirm causal relationships, temporal sequences, or true psychological mechanisms. All mediation effects reported in this study should be interpreted as statistical indirect associations rather than confirmed causal pathways. The theoretical sequential model proposed requires rigorous validation through longitudinal studies and randomized controlled trials.

Secondly, the representativeness and diversity of the sample still need to be further expanded. Although the sample of this study is relatively balanced in terms of basic demographic variables such as age, gender and geographical region, it did not control for factors such as the social economic status, educational level, type of physical exercise, and specific types and severity of history and chronic diseases of the older adults. To some extent, this may limit the general applicability of the research results.

Third, the reliance on self-report measures may introduce common method bias and subjective errors. All variables in this study were measured using self-rated scales. Although statistical tests indicated that common method bias was not severe, the influence of social desirability, recall bias, or current emotional states on responses cannot be entirely ruled out.

Fourth, the range of variables included in the model is limited and may have omitted other important mediating or moderating factors. This study focused on the serial mediating roles of self-efficacy and health anxiety. However, the psychosocial factors influencing aging anxiety are complex and multifaceted.

Fifth, while the statistical analyses confirmed the serial mediation effects, the exploration of group heterogeneity was insufficient. This study did not delve into whether the mediation model differs across subgroups with various demographic or health characteristics. Potential subgroup differences might obscure the unique psychological mechanisms operating within specific populations.

### 6.3 Future directions

Based on the findings and limitations of this study, future research could be further deepened and expanded in the following directions:

First, regarding research design, longitudinal tracking or experimental intervention designs could be considered to more accurately reveal causal relationships among variables. While this study preliminarily validated the serial mediation pathway using cross-sectional data, the causal directions still require further confirmation. Future research could employ long-term follow-up surveys to examine the dynamic interactions among physical exercise behavior, self-efficacy, health anxiety, and aging anxiety in older adults. Alternatively, randomized controlled trials could be designed to test the temporal effects of systematic physical exercise interventions on self-efficacy, health anxiety, and aging anxiety, thereby providing more robust causal evidence for the mental health benefits of physical exercise.

Second, in terms of research content, the boundary conditions and pathways of the mediating mechanisms could be further refined and expanded. Future studies could differentiate between types, intensities, and contexts of physical exercise to examine their varying impacts on aging anxiety and its psychological mechanisms. Additionally, other potential mediating or moderating variables could be explored, such as psychological resilience, social support, aging identity, and sense of meaning, to construct a more integrated theoretical model and comprehensively reveal the multi-path network through which physical exercise influences the mental health of older adults.

Third, concerning the research sample, it is recommended to enhance the diversity and representativeness of the sample to improve the external validity of the findings. Future studies could conduct cross-regional and cross-cultural comparative research to examine the moderating effects of socioeconomic and cultural factors within the model. Furthermore, in-depth analyses could be performed on specific subgroups of older adults with different health statuses, living arrangements, or levels of social participation to develop more targeted stratified intervention strategies.

Fourth, at the level of practical application, future research should focus on translating findings into practice by developing and validating evidence-based, structured physical exercise programs aimed at promoting mental health. Program design could integrate the serial mediation mechanisms revealed in this study, emphasizing the incorporation of psychological components for enhancing self-efficacy and managing health anxiety within exercise activities—such as goal setting, accumulation of successful experiences, and cognitive restructuring training—thereby achieving an intervention effect that integrates “body and mind.” Simultaneously, digital and intelligent delivery platforms could be explored to enhance the accessibility and sustainability of interventions, contributing to the realization of active aging and healthy aging.

## Data Availability

The raw data supporting the conclusions of this article will be made available by the authors, without undue reservation.
